# The effect of dietary supplementation with blueberry, cyanidin-3-O-β-glucoside, yoghurt and its peptides on gene expression associated with glucose metabolism in skeletal muscle obtained from a high-fat-high-carbohydrate diet induced obesity model

**DOI:** 10.1371/journal.pone.0270306

**Published:** 2022-09-16

**Authors:** Min Shi, Michael L. Mathai, Guoqin Xu, Xiao Q. Su, Andrew J. McAinch

**Affiliations:** 1 Institute for Health and Sport, Victoria University, Melbourne, Australia; 2 Australian Institute for Musculoskeletal Science (AIMSS), Victoria University, Melbourne, Australia; 3 Department of Sports and Health, Guangzhou Sport University, Guangzhou, China; Tohoku University, JAPAN

## Abstract

Obesity is a leading global health problem contributing to various chronic diseases, including type II diabetes mellitus (T2DM). The aim of this study was to investigate whether blueberries, yoghurt, and their respective bioactive components, Cyanidin-3-O-β-glucoside (C3G) and peptides alone or in combinations, alter the expression of genes related to glucose metabolism in skeletal muscles from diet-induced obese mice. In extensor digitorum longus (EDL), yoghurt up-regulated the expression of activation of 5’adenosine monophosphate-activated protein kinase (AMPK), insulin receptor substrate-1 (IRS-1), phosphatidylinositol-3 kinase (PI3K) and glucose transporter 4 (GLUT4), and down-regulated the expression of angiotensin II receptor type 1 (AGTR-1). The combination of blueberries and yoghurt down-regulated the mRNA expression of AGTR-1 and Forkhead box protein O1 (FoxO1) in the EDL. Whereas the combination of C3G and peptides down-regulated AGTR-1 and up-regulated GLUT4 mRNA expression in the EDL. In the soleus, blueberries and yoghurt alone, and their combination down-regulated AGTR-1 and up-regulated GLUT4 mRNA expression. In summary blueberries and yoghurt, regulated multiple genes associated with glucose metabolism in skeletal muscles, and therefore may play a role in the management and prevention of T2DM.

## Introduction

Skeletal muscle is the major site for uptake and storage of plasma glucose after a meal, and the process is mediated by the glucose transporter proteins glucose transporter 4 (GLUT4) and glucose transporter 1 (GLUT1) [1]. Insulin induces the translocation of GLUT4 from intracellular storage vesicles to the plasma membrane either by binding to its receptor, leading to increased receptor tyrosine kinase activity, phosphorylation of insulin receptor substrate-1 (IRS-1), or by downstream activation of the lipid kinase, phosphatidylinositol-3 kinase (PI3K) and the serine/threonine kinase Akt [2]. Apart from the insulin signalling pathway, an effective means to stimulate GLUT4 translocation to the plasma membrane is through the activation of 5’adenosine monophosphate-activated protein kinase (AMPK) [3]. Activation of AMPK has been viewed as a targeted approach to increase glucose uptake by skeletal muscle and control blood glucose homeostasis [4].

The extensor digitorum longus (EDL) is predominately a fast-twitch muscle, whereas the soleus is predominately a slow-twitch muscle [5]. Different fiber-types in these two muscles have shown different effects on insulin signal transduction pathway [6]. Slow-twitch oxidative skeletal muscle (the soleus) has greater insulin binding capacity as well as increased insulin receptor kinase activity and autophosphorylation compared with fast-twitch glycolytic skeletal muscle [7]. Furthermore, muscles with a greater percentage of oxidative myofibers have a higher content of GLUT4, which is associated with the improvement of glucose uptake [8].

Defects in the expression of critical elements of insulin signalling are known to cause insulin resistance in mammalian skeletal muscle, and these impairments in insulin action are associated with the development of prediabetes and type II diabetes mellitus (T2DM). Moreover, insulin resistance is associated with the renin-angiotensin system (RAS), in which the initial action of renin cleaves angiotensinogen to angiotensin I (ANG I), then ANG I converts to ANG II by the angiotensin converting enzyme (ACE) [9]. In skeletal muscle, ANG II, by acting on its receptor (AGTR-1), can induce insulin resistance by increasing cellular oxidative stress, leading to impaired insulin signalling and insulin-stimulated glucose transport activity [10]. Therefore, interventions that target RAS over activity, including ACE inhibitors and ANG II receptor blockers, are effective in ameliorating hypertension and improving whole-body and skeletal muscle insulin action.

Previous studies have shown an association between the consumption of fermented milk or yoghurt containing an abundance of lactic acid bacteria (LAB), and reduced obesity and T2DM [11]. The diet supplemented with yoghurt fermented by probiotic *Lactobacillus acidophilus* and *Lactobacillus casei* significantly delayed the onset of glucose intolerance, hyperglycemia, hyperinsulinemia, dyslipidemia, and oxidative stress in high fructose-induced diabetic rats, indicating a lower risk of diabetes and its complications [12]. Yoghurt is an excellent source of bioactive peptides, including antihypertensive peptides or ACE inhibitors [13, 14]. A recent study has shown that peptides with ACE inhibitory activity (derived from bovine α-lactalbumin) significantly reduced body weight, blood glucose and insulin levels, and down-regulated inflammation-related gene expression in adipose tissues of high-fat-diet (HFD)-fed C57BL/6J mice [15]. However, the mechanism triggered by yoghurt and its peptides involved in the regulation of glucose metabolism and improvement of insulin resistance is not fully elucidated.

Both *in vitro* and *in vivo* studies have suggested that consumption of blueberry products and their bioactive components (e.g.,C3G), have potential health benefits in regulating glucose metabolism and subsequently ameliorating the development of prediabetes and T2DM [16, 17]. Supplementation of 2% freeze-dried blueberry powder for 13 weeks in Obese Zucker rats have demonstrated significant reductions in glucose, fasting insulin and insulin resistance, as indicated by the Homeostasis Model Index of Insulin Resistance (HOMA-IR) [18]. Conversely, Prior et al. (2008) reported that long term supplementation with freeze-dried whole blueberry powder did not alter the glucose tolerance in C57BL/6J obese mice [19]. Likewise, our previous animal study also showed that blueberries did not improve glucose tolerance [20]. Thus the efficacy of blueberries on glucose metabolism is controversial and underlying mechanisms are not clear.

Cyanidin-3-O-β-glucoside (C3G) is a predominant bioactive anthocyanin compound found in many edible plants, for example blueberries, and has been reported to be protective against T2DM by attenuating multiple disorders *in vivo* and *in vitro* [21–24]. It has been found that C3G increased glucose uptake in human skeletal muscle cells due to its strong antioxidant activity [25]. C3G derived from black soybeans ameliorated T2DM in db/db mice potentially through inducing smaller insulin-sensitive adipocytes, which improve insulin signalling and increased glucose uptake [26]. It has also been demonstrated that C3G significantly induced AMPK activation and enhanced glucose uptake in L6 myotube cells [27]. However, the potential molecular mechanisms of C3G on glucose metabolism remain unclear.

We have previously reported that yoghurt and peptides with ACE inhibitory activity improve glucose clearance, as measured by a glucose tolerance test in the obese mouse model (C57BL/6) [20]. Therefore, due to the role of skeletal muscle in glucose regulation, in this study we aim to determine the impact of blueberries, yoghurt, C3G and yoghurt peptides, in isolation or combination, on mRNA expression of markers involved in glucose metabolism in two kinds of skeletal muscles, the EDL and the soleus in a diet induced obese mouse model. Understanding these changes in gene expression may lead to the development of effective therapeutic strategies for obesity and associated comorbidities such as T2DM.

## Material and methods

### Chemicals and reagents

iScript™ cDNA Synthesis Kit and SYBR Green Supermix were purchased from Bio-Rad Laboratories Pty Ltd (Gladesville, NSW, Australia). All other chemicals, unless otherwise specified, were obtained from Sigma-Aldrich Pty. Ltd. (Sunshine, VIC, Australia).

C3G (purity>97%) was provided by Polyphenols AS (Sandnes, Norway). Fresh blueberries (Rabbiteye blueberry) were obtained from Bhatti and Manj Australian Blueberries (Woolgoolga, NSW, Australia). Blueberries were characterised for their total anthocyanidins and cyanidin content using a reverse-phase HPLC [28]. The total content of anthocyanidins and cyanidinin dried blueberry powder were 1% and 0.31%, respectively. Cyanidin in anthocyanidins was 21.37%.

Skim milk was fermented by *Streptococcus thermophiles* ASCC 1275 and *Lactobacillus delbrueckii* subsp. *bulgaricus* 1466 to produce the yoghurt. The yoghurt was further fermented by *Lactobacillus helveticus* 881315 in the presence of Flavourzyme and the end product was used in the animal study. Peptides were extracted from yoghurt fermented by *Lactobacillus helveticus* 881315 in the presence of Flavourzyme for 12 hours. ACE inhibitory activity of peptides was determined using a reversed-phase-HPLC system (RP-HPLC, from Varian Analytical Instruments, Santa Clara, CA, USA) and the IC_50_ value of peptides was 1.47 ± 0.04 mg/mL. The details on the process of fermentation, peptide extraction and determination of ACE inhibitory activity have been reported previously [29]. Subsequently, peptides derived from the same batch (with an IC_50_ value of 1.47 ± 0.04 mg/mL) as we have previously reported were used in the present animal study [29].

### Animals and feeding regime

Six-week old male C57BL/6 mice were fed with a high fat diet (59% of total energy from fat) plus 30% fructose water for eight weeks to induce obesity and diabetes. Mice were then placed into divided cages (two in one cage) for dietary treatments for another eight weeks. Each mouse had individual *ad libitum* access to food and water during the experimental period. Experimental procedures were approved by the Animal Ethics Committee of Victoria University (AEC NO: 16/005).

The minimum number of animals (n = 5) in each treatment group was determined by a statistical power analysis (two tailed t-test at the 0.05 significance level) based on the power of 90% of expected changes in AMPK mRNA expression observed in a previous study [30], which is the primary outcome measured in this study. A conservative approach was then taken with an additional 5 animals added to account for a lower observed change or greater variance than predicted and any illnesses in animals. This study is a sub-study of the original study [20]. Eight treatment groups (n = 10 in each group) were included in the study: (1) standard chow with 5% fat (SCD), (2) high-fat high-carbohydrate diet (HFHC), and HFHC with six dietary supplementations including (3) blueberries (HFHC+B), (4) C3G (HFHC+C), (5) yoghurt (HFHC+Y), (6) peptides (HFHC+P)extracted from yoghurt, (7) combination of blueberries and yoghurt (HFHC+B+Y), and (8) combination of C3G and peptides (HFHC+C+P). The supplements were added to a mixture of gelatine. When it had set, the jelly supplement was then fed to mice separately to their HFHC diet. The dose of supplementations were 6.4 g/kg body weight (BW)/d of blueberries, 0.02 g/kg BW/d of C3G, 3 g/kg BW/d of yoghurt and 0.2 g/kg BW/d of peptides. The amount of supplements was chosen based on previous studies [20].

### Anaesthesia and skeletal muscle tissue collection

Following the eight week treatment period, the mice were deeply anaesthetised using isoflurane. Soleus and the EDL were collected into cryotubes and immediately frozen in liquid nitrogen for RNA analysis.

### Real-time polymerase chain reaction (PCR) analysis for skeletal muscle

RNA was extracted from the soleus and the EDL utilising a TRIzol based method according to the manufacturer’s instruction. Total RNA (0.5 μg) was reverse transcribed into cDNA using the iScript™ cDNA Synthesis Kit, according to manufactures instructions.

Oligonucleotide primers were designed using the Oligoperfect^™^ Suite (Invitrogen, Victoria, Australia) and were purchased from Integrated DNA Technologies, Inc. (Coralville, Iowa). The primer sequences used for the genes of interest are detailed in Table 1. ‘Real Time’ PCR was utilised using SYBR Green Supermix and MyiQ^™^ multiplex ‘real-time’ PCR detection system (Bio-Rad Laboratories, Hercules, CA). Relative changes in mRNA abundance was normalised to the average of the housekeeping gene, hypoxanthine phosphoribosyltransferase 1 (HPRT-1), then quantified using the 2^-ΔΔCT^method [31].

**Table 1:** Mouse primer sequences used for ‘Real Time’ PCR analysis of skeletal muscles.

Gene	Accession Number	Sequence
AGTR-1	NM_177322.3	Forward(5’—3’)TGGCTGGCATTTTGTCTGGAT Reverse(5’—3’)TGCTTTTCTGGGTTGAGTTGGT
AMPK-α	NM_178143.2	Forward(5’—3’) GCCCAGATGAACGCTAAGAT Reverse(5’—3’) TGCATACAGCCTTCCTGAGA
FoxO1	NM_019739.3	Forward(5’—3’)ACCCTGTCGCAGATCTACGA Reverse (5’—3’)AGGGACAGATTGTGGCGAAT
GLUT1	NM_011400.3	Forward(5’—3’)TTGCCCAGGTGTTTGGCTTA Reverse(5’—3’)GGCAGAAGGGCAACAGGATA
GLUT4	NM_009204.2	Forward(5’—3’)ACCAACTGGCCATCGTCATT Reverse(5’—3’)GGACAGAAGGGCAGCAGAAT
HPRT-1	NM_013556.2	Forward(5’—3’)GCAAACTTTGCTTTCCCTGG Reverse (5’—3’) ACTTCGAGAGGTCCTTTTCACC
IRS-1	NM_010570.4	Forward(5’—3’)TCCAGAAGCAGCCAGAGGAT Reverse(5’—3’)CGTGAGGTCCTGGTTGTGAA
PI3K	NM_001024955.2	Forward(5’—3’)TGATGTGGCTGACGCAGAAA Reverse(5’—3’)CCACGTCTTCTCGTCATGGT

AGTR-1, Angiotensin II receptor type 1; AMPK-α, 5’ AMP-activated protein kinase alpha; FoxO1, Forkhead box protein O1; GLUT1, Glucose transporter 1; GLUT4, Glucose transporter 4; HPRT-1, Hypoxanthine-guanine phosphoribosyltransferase;IRS-1, Insulin receptor substrate 1; PI3K, Phosphoinositide 3-kinase.

### Statistical analysis

Graph PadPrism Software 7.0 (GraphPad Software, Inc, La Jolla, CA, United States of America) was utilised for statistical analysis. All results were expressed as mean ± standard error of the mean (SEM) for each measurement (n = 8–10). One-way ANOVA was performed to analyse the significant differences in the mRNA expression of genes amongst treatment groups. Post-hoc analysis was conducted using Fisher’s (least significant difference; LSD) test for multiple comparisons amongst all groups. *P*<0.05 was considered significant.

## Results

### Intraperitoneal glucose tolerance test (ipGTT)

Following the 8-week supplementation, the blood glucose levels in the HFHC+C+P group and HFHC+Y at 30 and 60 min were significantly lower than those in the HFHC group (Table 2) [20]. The blood glucose levels in the HFHC+B group at 0, 30, 60 min and 120 min were significantly higher than those in the HFHC+Y and HFHC+P group [20]. In addition 8-week supplementations with yoghurt (HFHC+Y, 93.7 ± 5.3), peptides (HFHC+P, 98.4 ± 3.8) and the combination of C3G and peptides (HFHC+C+P, 97.2 ± 3.3) showed a significant improvement in the intraperitoneal glucose tolerance area under the curve (arbitrary units) compared to the HFHC group (117.2 ± 2.3), and the outcomes were comparable with that in the SCD group (97.0 ± 4.2) [20]. In contrast, supplementation with blueberries (HFHC+B, 127.2 ± 3.9), the combination of blueberries and yoghurt (HFHC+B+Y, 112.5 ± 2.3) and C3G group (HFHC+C, 103.1 ± 5.1) did not alter glucose tolerance compared with to the HFHC group [20].

**Table 2:** Changes in response to blood glucose level in diet induced obese mice with 8-week supplementation of blueberry, cyanidin-3-O-β-glucoside, yoghurt and its peptides.

	Blood glucose level (mmol/L)			
Treatment	0 (min)	30 (min)	60 (min)	120 (min)
SCD	11.5 ± 0.5	19.4 ± 1.0	14.9 ± 0.8	10.6 ± 0.6
HFHC	12.2 ± 0.3	24.5 ± 0.5 *	19.2 ± 0.8 *	11.9 ± 0.4
HFHC+B	13.8 ± 0.5	24.6 ± 0.5 *	21.6 ± 0.9 *	15.8 ± 1.2 *^#^
HFHC+Y	10.4 ± 0.4 ^a^	19.9 ± 1.2 ^#^ ^a^	14.4 ± 1.2 ^#^ ^a^	9.0 ± 0.7 ^a^ ^e^
HFHC+B+Y	12.1 ± 0.4	22.7 ± 0.5 *	18.4 ± 0.7 *^e^	12.5 ± 0.5
HFHC+C	10.6 ± 0.8	22.7 ± 0.9 *	15.7 ± 1.3 ^d^	10.8 ± 0.9 ^d^
HFHC+P	10.1 ± 0.6 ^b^	21.1 ± 0.8 ^b^	15.4 ± 0.8 ^#^ ^b^	9.4 ± 0.4 ^b^
HFHC+C+P	10.5 ± 0.5	20.3 ± 0.7 ^#^ ^c^	14.9 ± 0.8 ^#^ ^c^	9.9 ± 0.5 ^c^

SCD, standard chow; HFHC, high-fat high-carbohydrate diet; HFHC+B, HFHC with blueberries; HFHC+C, HFHC with C3G; HFHC+Y, HFHC with yoghurt; HFHC+P, HFHC with peptides extracted from yoghurt; HFHC+B+Y, HFHC with combination of blueberries and yoghurt; and HFHC+C+P, HFHC with combination of C3G and peptides.

Data were reported as mean ± SEM. Significant differences (p < 0.05) between groups are indicated as follows:

* SCD control vs the other groups;

^#^ HFHC vs supplemented groups;

^a^ represent HFHC+B vs HFHC+Y;

^b^ represent HFHC+B vs HFHC+P;

^c^ represent HFHC+B vs HFHC+C+P;

^d^ represent HFHC+B vs HFHC+C;

^e^ represent HFHC+Y vs HFHC+B+Y. Blood glucose level of others time points at 15 min, 45 min and 90 min are not shown and have previously been published [20].

### ‘Real-time’ PCR analysis of the expression of genes related to glucose metabolism in the EDL

**Fig 1:**
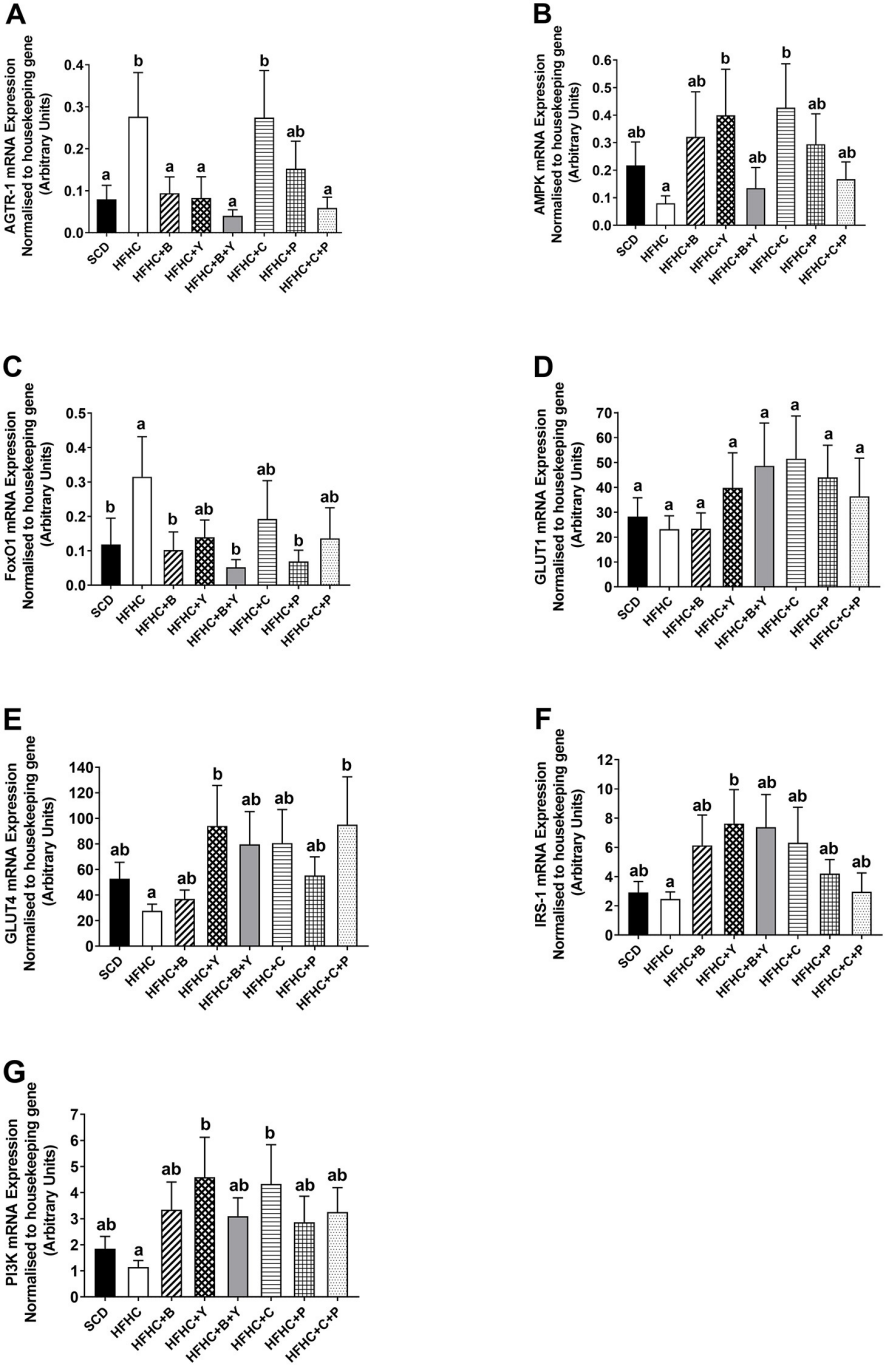
The expression of (A) AGTR-1; (B) AMPK; (C) FoxO1; (D) GLUT1; (E) GLUT4; (F) IRS-1 and (G) PI3K in the extensor digitorum longus (EDL) obtained from animals following various dietary treatments. Obese mice were treated with blueberries, C3G, yoghurt, peptides alone, and combinations of blueberries / yoghurt and C3G / peptides for eight weeks. All genes were normalised to the housekeeping gene, HPRT-1. SCD (n = 9), HFHC (n = 9), HFHC+B (n = 10), HFHC+Y (n = 10), HFHC+B+Y (n = 10), HFHC+C (n = 9), HFHC+P (n = 10), HFHC+C+P (n = 10). Data were expressed as mean ± SEM. Different letters indicate a significant difference between groups (P < 0.05).

Obese mice supplemented with blueberries and yoghurt, alone or in combination, as well as the combination of C3G and peptides in the EDL exhibited similar AGTR-1 expression to the SCD mice, and significantly lower AGTR-1 expression compared to HFHC (*P*<0.05; Fig 1A). While AGTR-1 expression was not changed by either C3G alone or peptides alone in the EDL compared to HFHC (Fig 1A). Mice supplemented with blueberries or combination of C3G and peptides showed a lower AGTR-1 expression than the C3G group (*P*<0.05; Fig 1A).

AMPK mRNA expression in EDL was significantly up-regulated in HFHC+Y and HFHC+C groups, compared with the HFHC group (*P*<0.05; Fig 1B).

The supplementation with blueberries and peptides alone, as well as the combination of blueberries and yoghurt significantly decreased FoxO1 mRNA expression in the EDL compared to HFHC (*P*<0.05; Fig 1C), and the level was comparable to that of SCD.

While GLUT1 mRNA expression was unaltered by any treatment (Fig 1D), yoghurt alone and the combination of C3G and peptides significantly increased GLUT4 mRNA expression in the EDL compared to HFHC (*P*<0.05; Fig 1E).

The expression of IRS-1 in the EDL was increased only following the yoghurt supplementation compared with the HFHC diet (*P*<0.05; Fig 1F).

Obese mice supplemented with yoghurt and C3G alone showed higher PI3K expression in the EDL compared to HFHC (*P*<0.05; Fig 1G).

### ‘Real-time’ PCR analysis of the expression of multiple genes related to glucose metabolism in the soleus

**Fig 2:**
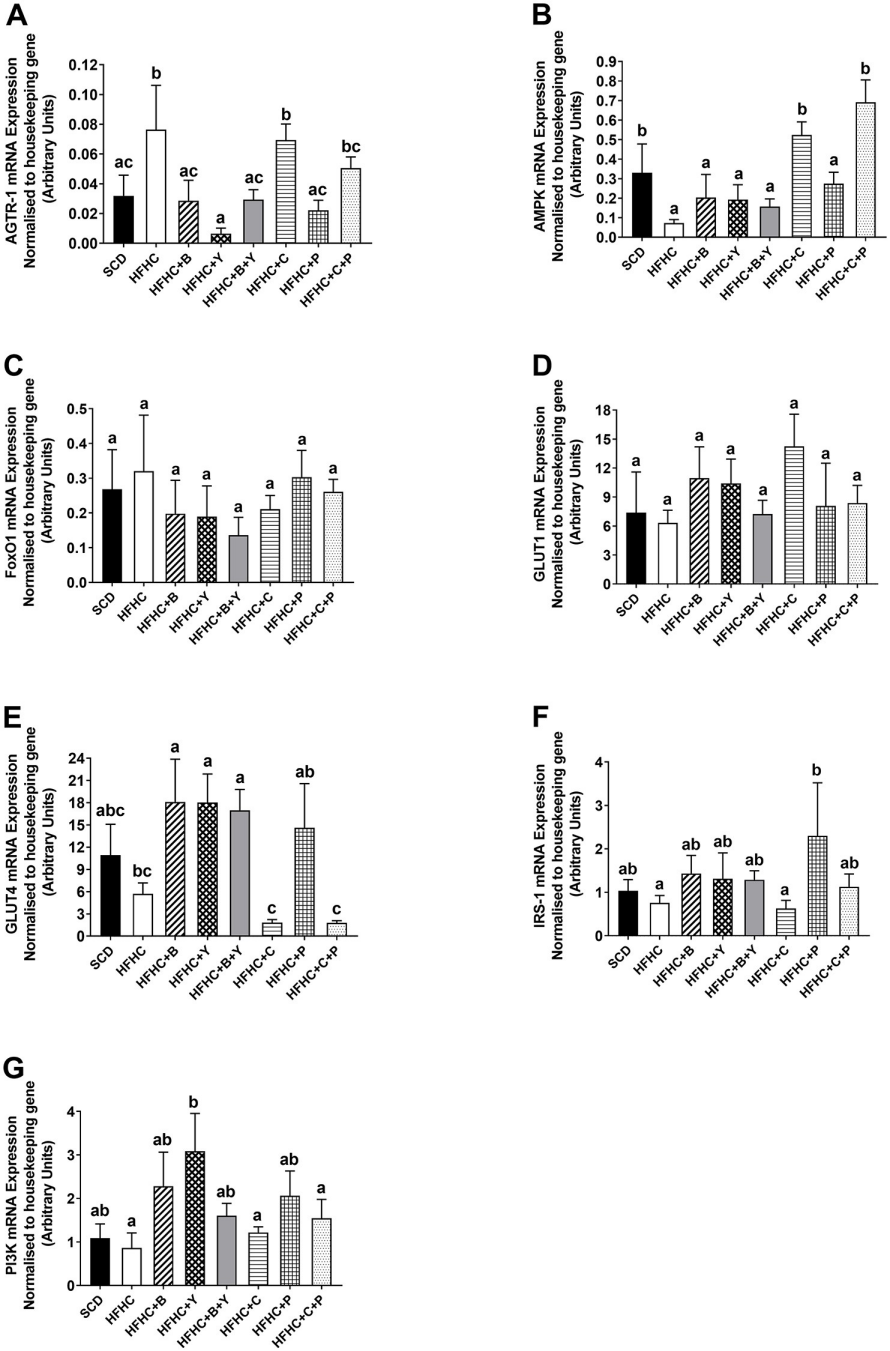
The expression of (A) AGTR-1; (B) AMPK; (C) FoxO1; (D) GLUT1; (E) GLUT4; (F) IRS-1 and (G) PI3K in the soleus obtained from animals following various dietary treatments. Obese mice were treated with blueberries, C3G, yoghurt, peptides alone, and combinations of blueberries / yoghurt and C3G / peptides for eight weeks. All genes were normalised to the housekeeping gene, HPRT-1. SCD (n = 10), HFHC (n = 9), HFHC+B (n = 10), HFHC+Y (n = 10), HFHC+B+Y (n = 10), HFHC+C (n = 10), HFHC+P (n = 10), HFHC+C+P (n = 10). Data were expressed as mean ± SEM. Different letters indicate a significant difference between groups (P < 0.05).

Four supplementations (HFHC+B, HFHC+Y, HFHC+B+Y and HFHC+P) significantly increased the mRNA expression of AGTR-1compared to the HFHC control group, while the other supplementations (HFHC+C and HFHC+C+P) did not cause alterations in the mRNA expression of AGTR-1 in the soleus compared to HFHC (*P*<0.05; Fig 2A). Moreover, blueberry supplementation resulted in a lower mRNA expression of AGTR-1 in the soleus when compared to C3G (*P*<0.05; Fig 2A).

The expression of AMPK in the soleus was significantly increased in mice supplemented with C3G and its combination with peptides compared to HFHC and other supplementation groups (*P*<0.05; Fig 2B).

The mRNA expression of FoxO1 and GLUT1 in the soleus was not altered by any treatment (Fig 2C & 2D).

A higher mRNA expression of GLUT4 in the soleus was observed following supplementation of blueberries, yoghurt and their combination compared with HFHC and C3G and its combination with peptides (*P*<0.05; Fig 2E), but not peptides alone.

The expression of IRS-1 in the soleus was increased only following peptide supplementation compared to HFHC (*P*<0.05; Fig 2F).

Yoghurt supplementation caused a significant increase in the mRNA expression of PI3K in the soleus compared to HFHC (Fig 2G).

## Discussion

Due to the important role of skeletal muscle in glucose regulation, this study focused on the impact of supplementation with blueberries and yoghurt, as well as some of their respective bioactive components on skeletal muscle mRNA expression of various genes related to glucose metabolism. This was the first study looking at the synergistic anti-diabetic effects of blueberries and yoghurt, as well as their bioactive components C3G and peptides on glucose metabolism in the EDL and the soleus skeletal muscle.

The major finding of this study was that eight weeks of supplementation with yoghurt increased the expression of multiple genes related to insulin-dependent (IRS-1/PI3K/GLUT4) and insulin-independent (AMPK) signalling pathways in skeletal muscle. These findings support our previous observation of yoghurt regulating glucose metabolism due to improved glucose tolerance in obese mice [20]. Moreover, these results were consistent with a recent study, which showed that a LAB, *Lactobacillus plantarum* Ln4 administration induced the up-regulation of hepatic mRNA levels, including IRS-2, Akt2, and AMPK, and subsequently improved systemic insulin resistance in mice [30]. Similar improvements in glucose metabolism via regulation of AMPK activity were also found following supplementation with fermented rice bran with fungi and LAB (*Lactobacillus brevis*, *Lactobacillus rhamnosus*, and *Enterococcus faecium*) in stroke-prone spontaneously hypertensive rats [32]. Moreover, treatment with an AGTR-1 blocker, telmisartan improved insulin sensitivity in obese db/db mice fed a high-fat diet [33]. Furthermore, *in vitro* treatment with telmisartan led to an increased level of AMPK phosphorylation, and an increase in the mRNA levels of GLUT4 in C_2_C_12_ myocytes [33]. Chronic administration of ACE inhibitors to insulin-resistant rodents has also been reported to increase protein expression of GLUT4 in skeletal muscle [34, 35]. Therefore the increased mRNA levels of AMPK and GLUT4 resulting from supplementation with yoghurt is possibly due to ACE inhibitory activity of peptides in the yoghurt.

It has been proposed that blockade of RAS may improve insulin sensitivity and prevent T2DM in metabolic tissues like liver, muscle and pancreas [36, 37]. A previous study showed that ANG II increased IRS-1 serine phosphorylation in vascular smooth muscle cells, and inhibited insulin-stimulated IRS-1 tyrosine phosphorylation, suggesting that ANG II might negatively modulate insulin-mediated actions by regulating multiple levels of the insulin signalling cascade such as the IR, IRS, and PI3K [38]. However, another study demonstrated that ANG II signalling contributed to glucose metabolism and inhibition of the insulin signalling pathway through AGTR-1 in both non-diabetic and diabetic vascular smooth muscle cells [39]. Furthermore, a recent study has shown that tripeptides with ACE inhibitory activity improved insulin resistance in rat-derived L6 skeletal muscle cells, at least partially via reduced AGTR-1 expression and attenuating reactive oxygen species (ROS) in L6 cells [40]. Yoghurt fermented by *Lactobacillus helveticus* (*L*. *helveticus*) includes bioactive peptides with a high ACE inhibitory activity, and tripeptides with ACE inhibitory activity have previously been shown to improve insulin resistance in L6 skeletal muscle cells, at least partially via reduced AGTR-1 expression and its anti-oxidative with reduction in reactive oxygen species (ROS) [40]. In the present study, yoghurt decreased AGTR-1 mRNA expression in both the EDL and the soleus of the obese mice possibly due to the effect of peptides with ACE inhibitory activity. This mechanism might explain the improved glucose tolerance observed in obese mice fed a diet supplemented with yoghurt [20].

*L*. *helveticus* is a LAB with a strong proteolytic system and subtypes of this family considered to be some of the most efficient strains in terms of production of anti-hypertensive peptides and aromatic compounds from caseins in fermented milk [41]. In the present study, peptides with ACE inhibitory activity extracted from yoghurt fermented by *L*. *helveticus* up-regulated IRS-1 and down-regulated AGTR-1 expression in the soleus. Consistently, the findings in our previous study have shown that these peptides could increase glucose uptake in human primary skeletal muscle myotubes [42] and improve glucose tolerance in obese mice [20]. Fructose-fed rats treated with Angiotensin-(1–7), an ACE inhibitor, exhibited an increased glucose uptake via a mechanism involved in the modulation of insulin signalling, through the IR/IRS-1/PI3K/Akt pathway in skeletal muscle, liver, and adipose tissue, as well as increased levels of IRS-1 phospho-Ser^307^ in skeletal muscle and adipose tissue [43]. Angiotensin-(1–7) also improved glucose uptake and decreased ROS production in 3T3-L1 adipocytes [44]. It has been reported that ANG II significantly decreased 5-aminoimidazole-4-carboxamide-1-beta-D-ribofuranoside (AICAR)-activated glucose uptake by the soleus muscles, and an AGTR-1 blocker cancelled the effect of ANG II, suggesting acute inhibition of the AGTR-1 improved glucose metabolism, not via an insulin pathway, but via an AMPK mediated pathway [45]. However, in the present study, peptides increased IRS-1 expression and reduced AGTR-1 expression but did not change AMPK expression in the soleus. Different animal models, treatment periods, different supplementations, and diet induced disease symptoms may contribute to the different results. It is still unclear how ANG II is involved in glucose metabolism, and further studies are needed to clarify the relationship among RAS, insulin pathway and AMPK pathway.

During milk fermentation, LAB hydrolyses lactose producing tagatose, which has low caloric value and is poorly degraded by the human body, making it an interesting anti-hyperglycemic agent [46]. Furthermore, an enzyme with ferulic acid esterase activity isolated from Lactobacillus johnsonii showed a potential effect on diabetes via stimulation of insulin production by ferulic acid and alleviation of symptoms caused by diabetes [47]. Food intake with weak organic acids, including lactic acid, formic acid, pyruvic acid and acetic acid, could increase insulin sensitivity and improve insulin resistance in T2DM by lowering interstitial fluid pH values [48]. *L*. *helveticus*has a strong proteolytic system, which is capable of producing not only short peptides, but also liberating amino acids from the casein matrix [49]. This system is composed of (i) cell envelope proteinases that hydrolyse caseins into oligopeptides, (ii) transport systems that allow uptake of oligopeptides, and (iii) various intracellular peptidases with differing and partly overlapping specificities, leading to a pool of free amino acids [50, 51]. Therefore, the anti-diabetic activity of yoghurt fermented by *L*. *helveticus* could not only be related to anti-hypertensive peptides, but also some bioactive proteins, organic acid enzymes and free amino acids produced during the fermentation by *L*. *helveticus* [48]. The results indicate that yoghurt and its bioactive components, including certain peptides play an important role in the regulation of glucose metabolism in muscle. Thus yoghurt is a potential therapeutic candidate in the prevention of T2DM through IR/IRS-1/PI3K/GLUT4, AMPK and AGTR-1 pathways.

In the present study, we also showed that blueberries down-regulated AGTR-1 expression in both the EDL and the soleus, and FoxO1 expression in the EDL. They also up-regulated GLUT4 expression in the soleus obtained from HFHC induced obese mice, although blueberries did not alter glucose tolerance in obese mice, as indicated in a previous study [20]. Two percent freeze-dried whole high-bush blueberry powder has been reported to increase the expression of IRS-1 and GLUT4 in the adipose and skeletal muscle tissues in both Zucker fatty rats and Zucker lean rats [52]. A recent study showed that blueberry supplementation improved markers of insulin sensitivity, including the normalized hepatic IRS-1 Ser^307^ phosphorylation and reduced hepatic malondialdehyde, a marker of oxidative stress in high-fat-diet–fed rats supplemented with blueberries [53]. However, neither blueberry supplementation nor C3G supplementation altered mRNA expression of IRS-1 in both skeletal muscle tissues in this study. Furthermore, fermented blueberry juice increased the phosphorylation of AMPK in C_2_C_12_ cells and 3T3-L1 cells, but treatment with non-fermented juice did not affect total AMPK content in either cell line [54]. Consistent with this, the present study also showed that blueberry supplementation did not alter mRNA expression of AMPK in either the EDL or the soleus. These inconsistent results in IRS-1 and AMPK in the current study and those in the literature may be due to the type of tissues analysed or animal species.

In order to determine which components in blueberries showed the major bioactivity on the regulation of genes related to glucose metabolism, C3G, a typical anthocyanin in blueberries was also investigated in this study. Several studies have shown that C3G regulates glucose metabolism via stimulating AMPK activation in skeletal muscle and high glucose-incubated adipocytes, as well as via attenuating high-glucose-promoted O-glycosylation of transcription factor FoxO1 in 3T3-L1 adipocytes [23, 55, 56]. These findings were in agreement with the present study, in which C3G was found to increase the mRNA expression of AMPK in both the EDL and the soleus, and PI3K in the EDL. However, C3G did not improve glucose tolerance in obese mice [20], suggesting that the up-regulation of AMPK and PI3K mRNA in skeletal muscle does not appear to be sufficient to influence glucose metabolism in this model. Similarly C3G in isolation or in combination with peptides (as used in these current study), resulted in an up-regulation in the mRNA expression of AMPK and PI3K in human primary skeletal myotubes [42]. These findings were in agreement with the present study, in which C3G was found to increase the mRNA expression of AMPK in both the EDL and the soleus, and PI3K in the EDL. Previous reports have shown that C3G increases GLUT4 membrane translocation and the increase of GLUT4 expression in murine adipocytes 3T3-L1, as well as through the regulation of GLUT4-retinol binding protein (RBP4) system [16, 24]. Therefore, blueberry and C3G regulate key molecules related to glucose metabolism potentially through the AMPK and PI3K/AKT/GLUT4 pathways as well as the inhibition of AGTR-1 and FoxO1 expression, although these effects on cellular mechanisms appears to not be large enough to improve glucose tolerance in obese mice [20]. Interestingly, the two combination treatments (HFHC+B+Y and HFHC+C+P) did not appear to show an additive effect on the regulation of multiple genes related to glucose metabolism in the skeletal muscle of obese mice. Further investigation is required to determine the possible interactions of various bioactive components in blueberries and yoghurt.

## Conclusions

Supplementation with blueberries resulted in a reduction of the mRNA expression of AGTR-1 in the EDL and the soleus. However, a key bioactive component of blueberries, C3G displayed a role in the up-regulation of the mRNA expression of AMPK in both the EDL and the soleus suggesting that the anti-diabetic mechanism of blueberries was different from that of C3G. Thus the potential anti-diabetic properties of blueberries may be related to bioactive components other than C3G, or the synergistic effects of C3G and those components. Furthermore, yoghurt showed a potentially anti-diabetic activity involving an insulin-dependent signalling pathway associated with an increase in the mRNA expression of PI3K, IRS-1 and GLUT4; and an insulin-independent signalling pathway associated with an increase in the mRNA expression of AMPK and a decrease in the mRNA expression of AGTR-1. The different muscle tissues examined, which have different muscle fiber type composition, responded differently to the various dietary interventions, some of which is thought to be due to their oxidative capacity and substrate preferences. The exact reason for these different responses are however difficult to elucidate. Understanding further the molecular mechanisms responsible for these different responses and their roles in glucose regulation should be the focus of future studies. A limitation of this study is that the protein expression/activity of the genes concerned was not measured. Future investigation on these aspects as well as through human clinical trials will help to further understand the molecular mechanisms underlying the health effects particularly of yoghurt and its bioactive peptides on glucose metabolism.
